# Source apportionment of chlorinated polycyclic aromatic hydrocarbons associated with ambient particles in a Japanese megacity

**DOI:** 10.1038/srep38358

**Published:** 2016-12-06

**Authors:** Yuta Kamiya, Akihiro Iijima, Fumikazu Ikemori, Tomoaki Okuda, Takeshi Ohura

**Affiliations:** 1 Graduate School of Agriculture, Meijo University, 1-501 Shiogamaguchi, Nagoya 468-8502, Japan; 2 Faculty of Regional Policy, Takasaki City University of Economics, 1300 Kaminamie, Takasaki 370-0801, Japan; 3 Nagoya City Institute for Environmental Science, 5-16-8 Toyoda, Nagoya 457-0841, Japan; 4 Department of Applied Chemistry, Faculty of Science and Technology, Keio University, 3-14-1 Hiyoshi, Kohoku-ku, Yokohama 223-8522, Japan

## Abstract

Chlorinated polycyclic aromatic hydrocarbons (ClPAHs) are novel species of environmental contaminants whose possible sources remain unclear. The occurrence of ClPAHs within total suspended particles (TSP) is compared with weekly air samples at two sites of differing characteristics (industrial and residential) in the megacity of Nagoya, Japan. Samples were collected over 12 months during 2011–2012. All 24 species of targeted ClPAHs were detected at both industrial and residential sites, where mean concentrations of total ClPAHs in TSP were 20.7 and 14.1 pg/m^3^, respectively. High concentrations at the industrial site were frequently observed during winter, suggesting potent seasonal ClPAH sources there. Positive matrix factorization modeling of particulate ClPAH source identification and apportioning were conducted for datasets including ClPAHs, PAHs, elements and ions, plus elemental carbons in TSP. Eight factors were identified as possible ClPAH sources, with estimates that the dominant one was a specific source of ClPAH emission (31%), followed by traffic (23%), photodegradable and semi-volatile species (18%), long-range transport (11%), and industry and oil combustion (10%). Source contributions of ClPAHs differed substantially from those of PAHs. This suggests specific and/or potent ClPAH sources in the local area, and that the production mechanisms between ClPAHs and PAHs are substantially different.

Polycyclic aromatic hydrocarbons (PAHs) have been widely recognized as very hazardous air pollutants that must be addressed by the international community, because of strong toxicity such as mutagenicity and carcinogenicity, plus substantial production by various natural and anthropogenic sources^1^,^2^,^3^,^4^,^5^. Therefore, studies of ambient PAHs have covered broad areas such as analysis of environmental concentrations and behaviors, including photochemical reactions and gas/particle partitioning, predictions of potential sources, and evaluation of exposure risks^2^,^5^. It is known that atmospheric PAHs are mainly produced by incomplete combustion of organic substances^1^. In particular, industrial plants such as coke and aluminum production, residential heating, and automobiles constitute the majority of anthropogenic PAH sources. In addition to these local sources, regional effects such as long-range transport (LRT) and volatilization of previously deposited PAHs from surfaces could be important to ambient PAH concentrations. The contributions of such potential sources to ambient PAH concentrations vary significantly by area and season; therefore, identification of those sources is necessary for PAH reduction and control in the environment, toward a reduction of exposure risk.

The sources of environmental PAHs have been estimated by several methods. Diagnostic ratio analysis is effectively used as a simple and common method for identification, although it is a qualitative assessment^6^. In contrast, principal component analysis, chemical mass balance (CMB), and positive matrix factorization (PMF) using statistical receptor models have been used in quantitative source contribution analyses. In particular, PMF is an acceptable tool to apportion sources as compared with CMB, which requires source profiles. PMF has been effective for source identification and apportionment of ambient PAHs around the world^7^,^8^,^9^,^10^,^11^,^12^.

The chlorinated PAHs (ClPAHs) are of increasing interest in the study of PAH derivatives as new classes of environmental contaminants, because findings indicate strong environmental persistence and toxicities similar to PAHs and dioxins^13^,^14^. Study involving surveys of ClPAH in the environment has been advanced by atmospheric sampling, which has been done in urban and suburban cities in Japan^15^,^16^,^17^,^18^,^19^, Sweden^20^, South Korea^21^, and China^21^,^22^. Significant correlations of concentrations between ClPAHs and corresponding parent PAHs have been commonly found. It is therefore believed that ClPAHs have sources similar to PAHs. Thus, as a next step in source identification, diagnostic ratios of typical ClPAHs have been assigned and compared with those from possible source samples such as traffic exhaust and fly ash^17^,^23^,^24^. Although this approach may be appropriate for rough understanding of the involvement of sources, identification was limited and quantitative evaluation impossible.

In the present study, we investigated the occurrence of ClPAHs and PAHs using preferential source markers such as ions, elements, organic carbon, and elemental carbons in total suspended particles (TSP) at two sites over a year. These were industrial and residential sites in the megacity of Nagoya, Japan (Figure S1 of the Supplementary Information). Furthermore, ClPAH source apportionment associated with particles was done by PMF with the obtained datasets, and we addressed the characteristics of estimated sources. This is an initial report investigating specific ClPAH sources in the atmosphere.

## Results and Discussion

### Occurrences of ClPAHs in TSP

**Figure 1:**
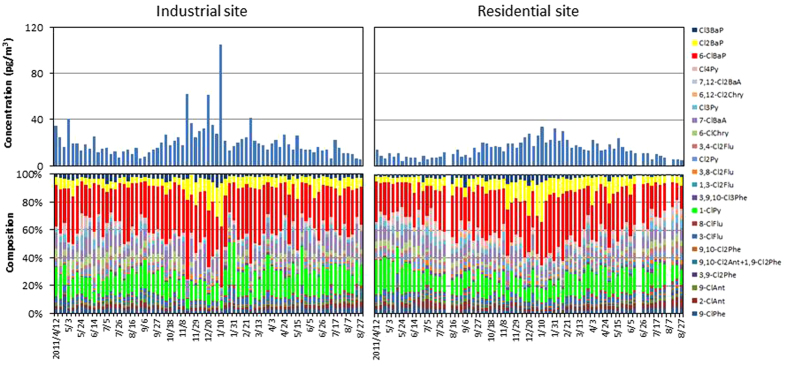
Weekly variation of total ClPAH composition at industrial (left) and residential (right) sites.

All 24 species of ClPAHs (Figure S2) were detected in TSP samples during campaigns at both sites. The mean, standard deviation, minimum and maximum concentrations, and industrial/residential site concentration ratio (I/R) of each compound are summarized in Table S1 of the Supporting Information. Mean concentrations of total ClPAHs at the industrial and residential sites were 20.7 and 14.1 pg/m^3^, respectively, so the concentration at the industrial site was 1.5 times higher than that at the residential site. Those mean concentrations are similar to those in urban air at Shizuoka, Japan (17 pg/m^3^)^18^, but tended to be more than twice those in other urban cities of Japan^21^. Throughout the sampling period at both sites, concentrations of total ClPAHs tended to increase in winter and decrease in summer (Fig. 1). Indeed, significant negative correlations (*p* < 0.01) were observed between ambient temperature and total ClPAH concentrations at both sites (data not shown). This variation of concentration driven by temperature has been observed in other works^16^,^17^,^18^, suggesting that atmospheric ClPAH concentration is influenced by several seasonal factors. These factors include atmospheric loss mechanisms such as gas-particle distribution and photodegradation, and seasonal emission sources such as heating systems. A sudden increase of total ClPAH concentrations was observed at the industrial site from November 2011 to January 2012, but such change was not evident at the residential site (Fig. 1). There were no significant differences of total ClPAH concentration between the two sites in summer. However, from winter to spring, total concentrations at the industrial site tended to be higher than at the residential site. The temporal and spatial distributions of air pollutants may be driven not only by characteristics of emission sources but also various atmospheric physicochemical processes such as transport, gas-particle portioning, atmospheric reactions by radicals and oxidants, and dry and wet deposition. In particular, such atmospheric processes possibly occur during LRT^25^. Here, we investigated the effect of air mass transport on the increased ClPAH concentration at the industrial site, using the Hybrid Single Particle Lagrangian Integrated Trajectory model. This indicated similar trajectories around the highest concentration event (data not shown). This implies that characteristics of emission sources rather than LRT affect the local ClPAH concentration.

Compositions of individual ClPAHs in TSP showed specific distributions for each site and season (Fig. 1). 6-ClBaP was dominant in all ClPAHs detected, and its compositions at the industrial and residential sites were 32% (range 18–57%) and 28% (12–49%), respectively. The second largest contributor was 1-ClPy, compositions of which were 16% (6–31%) at the industrial site and 15% (8–25%) at the residential site. Such orders of ClPAH magnitude have been observed not only for particles in urban air^16^,^17^ but also in sediments^26^,^27^ and fly ash samples of waste incineration^23^. However, those in particles in urban streets and road tunnels showed a very different pattern, in which Cl_2_Py and 1-ClPy were relatively abundant components in a total of nine targeted ClPAHs^20^. In our study, for overall patterns, compositional variation was greater at the industrial site than the residential site. In particular, contributions of 6-ClBaP at the industrial site increased in the cold season as compared with the residential site. Additionally, periods of substantial 6-ClBaP contributions closely fit those with drastic increase of total ClPAH concentrations at the industrial site (Fig. 1). As described above, the event with higher ClPAH concentration may be associated with local sources. Thus, 6-ClBaP could be a major contributor among ClPAHs in the atmosphere, possibly pointing to local industrial activities.

### Occurrences of PAHs in TSP

We detected all 22 PAHs (Figure S3) in TSP samples collected at both sites. Mean concentrations of total PAHs at the industrial and residential sites were 3.1 and 1.9 ng/m^3^, respectively (Table S2). The concentration of total PAHs at the industrial site was 1.6 times higher than at the residential site, similar to the case of ClPAHs. In addition, the total PAH concentration was approximately three orders of magnitude higher than that of ClPAHs at both sites. Such a magnitude difference was also observed in a past study^19^. In general, concentrations of particulate PAHs are known to increase in winter and decrease in summer. However, in the present study, the concentrations showed no such distinct seasonal variation and a pattern very distinct from that of ClPAHs (Figure S4). It is believed that the generalized seasonal trend of PAHs is attributable to the influences of seasonal factors such as gas-particle partitioning, photodecay reactions by sunlight, and seasonal heating sources^1^. Thus, the current PAHs in TSP may have been continuously emitted by local sources such as automobile exhaust, which is independent of season.

Comparing the composition of total PAHs, similar compositions were observed throughout the monitoring period at both sites (Figure S5). This indicates that the sources of PAHs in TSP were equally present at the sites. This finding clearly differs from the ClPAH composition patterns, suggesting that this difference is caused by the presence of specific sources of ClPAHs, but not of PAHs.

### Occurrence of carbons, ions, and elements in TSP

Carbons, ions and elements in TSP were also determined simultaneously, to evaluate factors affecting ClPAH concentrations. Mean concentrations of OC and EC were respectively 4.3 and 2.0 μg/m^3^ at the industrial site, and 3.6 and 1.3 μg/m^3^ at the residential site (Table S3). Both carbon concentrations were slightly (1.1–1.6 times) higher at the industrial site than at the residential site. Although EC temporal variations were relatively constant throughout the sampling period at both sites, those of OC had a specific pattern, i.e., greater in spring and autumn than in other seasons (Figure S6). The seasonal increase of OC may be attributable to spring storms, i.e., yellow dust and formation of secondary organic aerosol.

Among water-soluble ions, the mean concentration of sulfate ions was the highest throughout the period, followed by nitrate and ammonium ions at both sites (Table S4). Interestingly, temporal trends of sulfate ions showed a specific variation relative to nitrate and ammonium ions (Figure S7). The high concentrations of sulfate ions in the warm season are likely attributable to local industrial activities in addition to secondary formation.

Regarding elements, the abundance of concentration varied greatly for each component, and mean concentrations of individual elements were higher at the industrial site than at the residential site (Table S5). There was irregular temporal variation of each element, not the regular seasonal variation, suggesting that they are related to local sources rather than LRT (Figure S8).

### Relationships of ClPAHs and PAHs

**Figure 2:**
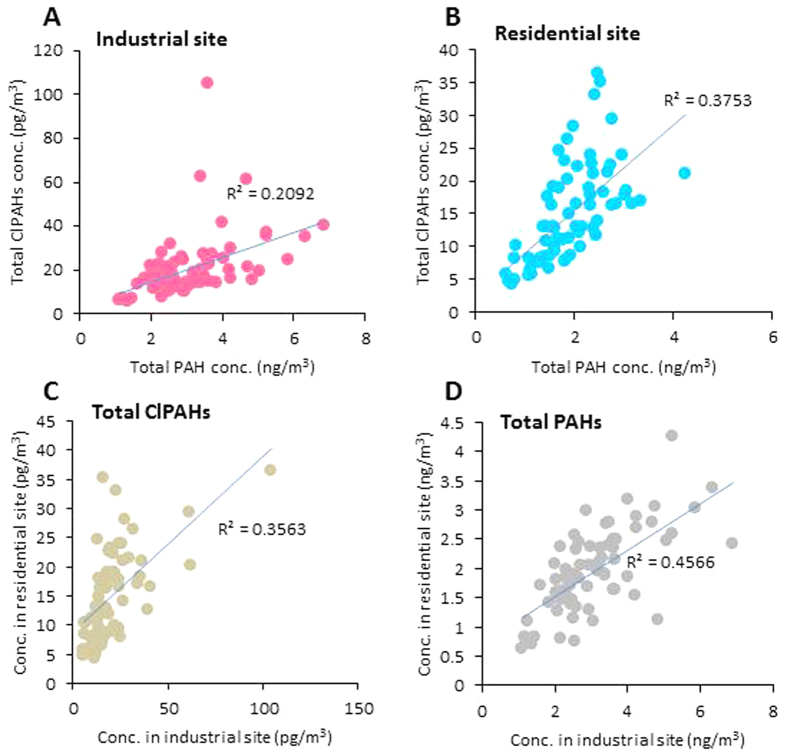
Relationships of concentrations between total ClPAHs and total PAHs at industrial site (**A**) and residential site (**B**) in TSP. Relationships of total ClPAH (**C**) and total PAH (**D**) concentrations in TSP between industrial and residential sites.

Correlation analysis determines commonality, so it is a common technique to evaluate emission sources of various pollutants. First, we investigated the relationship between total PAH and total ClPAH concentrations at each sampling site. In the TSP samples, significant correlations (*p* < 0.01) were observed at both sites (Fig. 2A and B). These results indicate that the production of ClPAHs is irrefutably related to those of PAHs. However, as noted above, significant variations of concentrations and compositions were observed in ClPAHs but not in PAHs. These tendencies were more pronounced at the industrial site. Indeed, comparing the relationship of ClPAH or PAH concentrations between the two sites, correlation of total ClPAH concentrations (Fig. 2C) was weaker than that of total PAH concentrations (Fig. 2D). Furthermore, to investigate commonality of individual ClPAHs or PAHs, we analyzed correlation among individual ClPAHs or PAHs at the industrial site. Concentrations of most PAHs in TSP showed significant correlation (*p* < 0.05) with each other (Table S6 shows an example for the industrial site), but there was weaker correlation for ClPAHs (Table S7). This suggests that almost all PAHs associated with particles originate from common sources in the area, whereas certain ClPAHs are strongly influenced by different emission strengths of their common sources and the presence of specific sources.

### Source apportionment

Table S8 lists the input data statistics. An eight-factor solution was resolved by PMF as an optimum solution. *Q*/*Qexp* and *r*^2^ indicated that all the samples and species were well modeled. The scaled residuals were roughly symmetrically distributed between −3 and +3. We conducted 100 bootstrap (BS) runs with a minimum correlation value of 0.6. The boot factors were uniquely matched with the base factors. Although several values for Fpeak were tested, altering the Fpeak value did not result in substantially better source profiles. The results of error estimation (BS, bootstrap-displacement (BS-DISP), and displacement (DISP)) for TSP mass concentration and specific elements are summarized in Table S9 and Table S10, respectively. The characterizations of each factor are explained with the following paragraph. The results of error estimation are stable as indicated by the three uncertainty tools. Consequently, we accepted the base run result as a final solution.

**Figure 3:**
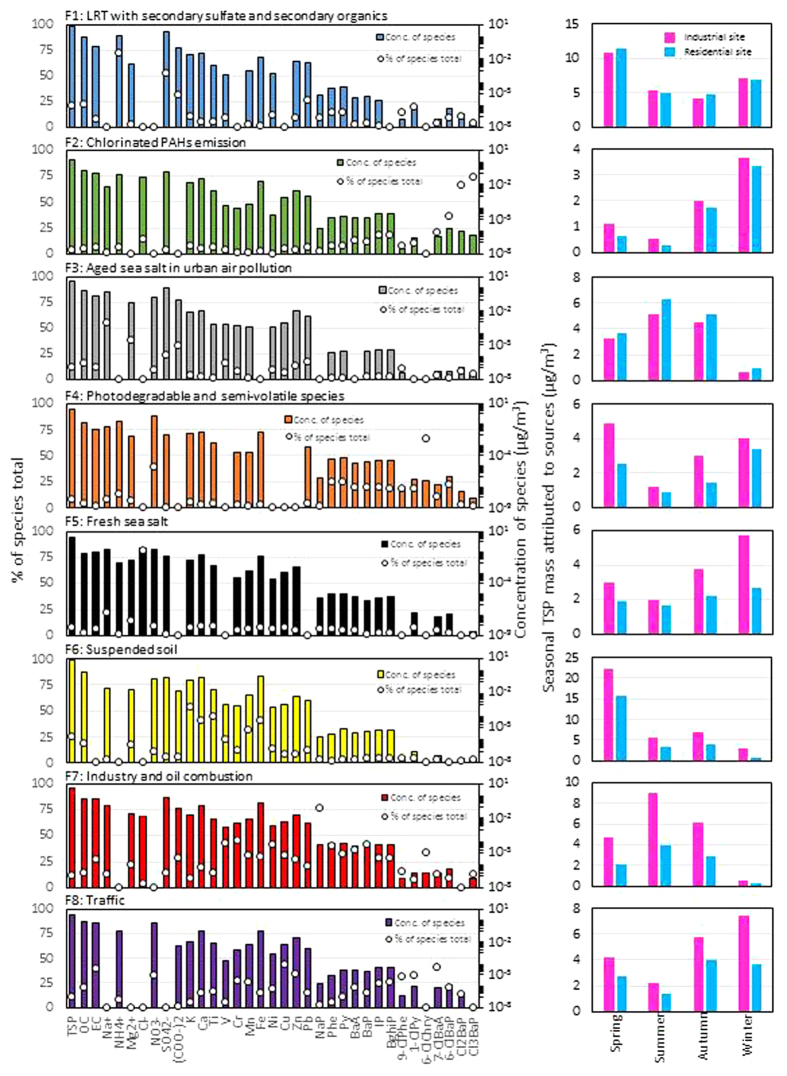
PMF factors profiles for the identified sources (left) and the estimated seasonal concentrations of TSP (right).

Figure 3 depicts resolved factor profiles and seasonal associated TSP contributions for the eight-factor solution. Also, the weekly contributions of each factor are represented in Figure S9. Factor 1 (F1) was made up of SO_4_^2−^, NH_4_^+^, and (COO)_2_^2−^ (Fig. 3), and thus clearly represents secondary generation^28^,^29^. The contributions of F1 at the industrial and residential sites were comparable, and typically increased from winter to spring, during which there were strong westerly winds. Therefore, F1 is attributed to widespread pollution caused by LRT with secondary sulfate and secondary organics. Factor 2 (F2) represents ClPAH emission, and its contribution at the industrial site was slightly greater than at the residential site. The contribution of F2 increased in winter, when atmospheric stability is strong. It is difficult to identify the potential sources corresponding to F2, but industry-related ClPAH sources have been suggested. Comparison between measured profiles and emission inventory data will become an issue in the future. Factor 3 (F3) had strong contributions of Na^+^, Mg^2+^, and shares of SO_4_^2−^ and (COO)_2_^2−^. It is well known that sea salt particles are impacted in the atmosphere by pollutants, with loss of particulate chlorine to the gaseous phase^30^. Chlorine loss is usually attributed to ion exchange reactions with SO_2_, H_2_SO_4_ and HNO_3_, with the formation of sulfates and nitrates. Equivalent concentrations of cations (Na^+^ and Mg^2+^) and anions (NO_3_^−^ and SO_4_^2−^) were in good agreement in the F3 profile. The contribution of F3 tended to increase in the warm season with high relative humidity, strong photochemical activity, and heavily polluted atmosphere (details are in Factor 6). Moreover, that contribution at the residential site (in an inland area ~10 km from the coastline, Figure S1) was slightly greater than at the industrial site (in a coastal area), probably because of the difference in residence time of sea salt particles. Therefore, F3 represents aged sea salt in urban air pollution. Factor 4 (F4) is composed of nitrate and combustion tracers of PAHs. A somewhat greater contribution was observed at the industrial site, with massive anthropogenic emission sources. The contribution of F4 clearly decreased in summer because of the semi-volatile and photodegradable characteristics. This factor corresponds to physicochemical characteristics rather than specific emission sources. For this reason, F4 was recognized as photodegradable and semi-volatile species. Factor 5 (F5) is characterized by Cl^−^ and weak Na^+^, which are clearly associated with fresh sea salt. A greater contribution was observed at the coastal industrial site than at the inland residential site. At the former site, the contribution tended to increase in winter because of strong wind. However, there was no clear seasonal variation at the residential site. This difference could be due to the distance between the site and sea. Factor 6 (F6) is dominated by elements such as K, Ca, Ti, Mn and Fe, which are tracers of soil/crustal sources^31^. Large contributions of F6 observed in spring are caused by the suspension of wind-blown soil dust and Asian dust storms. Thus, F6 can be defined as suspended soil. Factor 7 (F7) is enriched with anthropogenic-related elements and PAHs. It is reasonable that greater contributions were observed at the industrial site. Notably large shares of V, Ni, and PAHs suggest the contribution of oil combustion^32^. In summer, the sites are dominated by southerly breezes (2.6–3.5 m/s on average) from the nearby sea, Ise Bay (Figure S1), near which there is a large petrochemical complex and vessels operating. On the other hand, the winds (2.6–3.1 m/s on average) over the sites in winter were mostly blowing from west-northwest. Therefore the sites in winter could be less affected by the sources presented in seaside (Figure S1). Also, the seasonal trend of F7 is very similar to that of F3. This supports the hypothesis of the activation of ion exchange reactions between sea salt particles and a polluted atmosphere. Therefore, F7 represents industry and oil combustion. Factor 8 (F8) can be characterized by anthropogenic-related species such as OC, EC, NO_3_^−^, Cu, Zn, PAHs, and two ClPAHs. The contribution of F8 was greater at the industrial site, and increased in the cold season with its strong atmospheric stability. It is known that Cu, Zn, and PAHs are traffic-related pollutants (brake wear, tire wear, and exhaust, respectively). Moreover, the OC/EC ratio in the F8 profile is consistent with vehicle exhaust particles. All the above considered, F8 may correspond to traffic.

**Figure 4:**
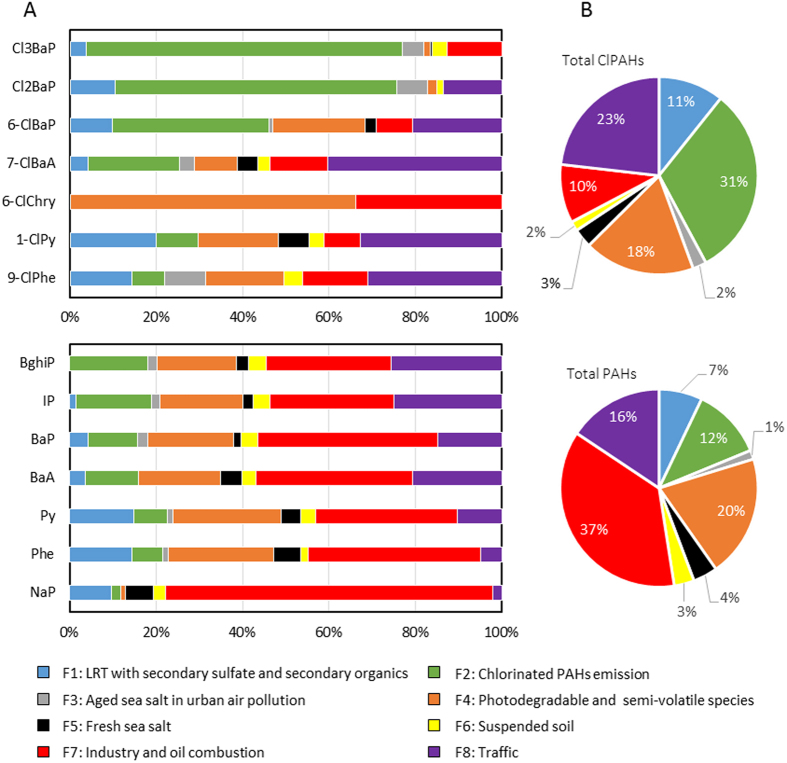
Source contributions of individual (**A**) and total concentrations (**B**) of ClPAH and PAH for associated particles.

Next, we estimated the contributions of potential sources to individual ClPAHs and PAHs, and total concentrations (Fig. 4). Note that the relationship between measured and modeled total ClPAHs (7ClPAHs used in the PMF analysis) concentrations was significant (*R*^2^ = 0.62, *p* < 0.01) and the slope of the regression line (0.71) was close to unity indicating a close agreement (Figure S10). The major contributor to total ClPAH concentrations was 31% for ClPAH emissions (F2), followed by 23% for traffic (F8) and 18% for photodegradable and semi-volatile species (F4) (Fig. 4B). In contrast, the dominant source of total PAH concentration was industry and oil combustion (F7) at 37%, followed by photodegradable and semi-volatile species (F4) at 20%, and traffic (F8) at 16% (Fig. 4B). Interestingly, the contributions of ClPAH emissions (F2) and industry and oil combustion (F7) showed very different rates between ClPAHs and PAHs (Fig. 4B). This suggests the following possibilities: (i) Specific and/or potent sources for ClPAHs or PAHs are present in the local area, (ii) the production mechanisms of ClPAHs and PAHs are very different. Furthermore, the contributions of F2 increased with the molecular weight of ClPAH, whereas those of F2 for PAHs did not show such variation (Fig. 4A). As shown in Fig. 3, it was found that the contributions of F2 were elevated in the cold season. However, we could not obtain more information on F2. Identification of the source of F2 would provide valuable information for the understanding of production mechanisms of ClPAHs. Regarding traffic (F8) as a source, there were somewhat consistent contributions of both total ClPAHs and total PAHs, between 16% and 23% (Fig. 4B). Traffic exhaust is known to be one of the potent sources of ambient PAHs^1^. We also recently detected numerous ClPAH species from tunnel dust^33^. These findings suggest that traffic exhaust control contribute substantially to the reduction of those compounds in the environment. For all ClPAHs and PAHs, the contributions of aged sea salt (F3), fresh sea salt (F5), and suspended soil (F6) were at an almost constantly small rate, 7–8% of the total (Fig. 4B).

In conclusion, source contributions of ClPAHs differed from those of PAHs. In particular, the specific source of ClPAHs was newly discovered by the PMF analysis. Although the specific source may be strongly driven by seasonal factors, detailed information remains unclear. Further studies on ClPAHs must have data on their specific sources. In addition, if gaseous ClPAHs are simultaneously measured, the derived source model could enhance accuracy and be extended to understand ClPAH formation and perform assessments.

## Methods

### Chemicals and reagents

Twenty-four ClPAH species with three to five rings were targeted. Structures and abbreviations of those ClPAHs are presented in Figure S1. These parent PAHs (skeleton PAHs; phenanthrene, anthracene, fluoranthene, pyrene, chrysene. benz[*a*]anthracene, and benzo[*a*]pyrene) were chosen for toxicological preference and environmental universality. Detailed information, including ClPAH synthesis methods of, has been reported elsewhere^16^. In addition, 22 PAH species, including 16 of US Environmental Protection Agency priority, were targeted. These were purchased from AccuStandard Inc. (Connecticut, USA), and their structures and abbreviations are presented in Figure S2. Deuterated PAHs used as internal standards were phenanthrene-d_10_ (Phe-d_10_), fluoranthene-d_10_ (Fluor-d_10_), and perylene-d_12_ (Pery-d_12_), which were purchased from Cambridge Isotope Laboratories Inc. (Andover, Massachusetts, USA). For analysis of inorganic compounds, we used SO_4_^2−^, NO_3_^−^, Cl^−^ and (COO)_2_^2−^ as anions and Na^+^, NH_4_^+^, K^+^, Mg^2+^ and Ca^2+^ as cations. Each standard mixture solution was acquired from Wako Pure Chemicals Industries, Ltd. (Osaka, Japan). Solvents used for extraction and cleanup were residual pesticide analysis grade (>99.5% by GC, also from Wako Pure Chemicals or Kanto Chemicals).

### Atmospheric sampling

TSP was simultaneously sampled at the industrial and residential sites in Nagoya, from April 2011 through August 2012. The city of Nagoya is near the center of Japan and has ~2.3 million inhabitants, making it the third largest city in the country (Figure S1). Its southern portion is on the sea and is one of the large-scale industrial areas in Japan, where there is a wide variety of industrial plants. Sampling at the industrial site was done at rooftop locations (20 m above ground level) at the Nagoya City Institute for Environmental Science (35°5′N, 136°54′E), ~100 m from the nearest busy road. Sampling at the residential site was also done at rooftop locations (20 m above ground level), on the Meijo University campus (35°8′N, 136°58′E). TSP samples were collected on 20.3- × 25.4-cm quartz fiber filters (QFFs; Tokyo Dylec Co., Ltd., Tokyo), weights of which were determined by a high-volume air sampler (HV-700F; Sibata Scientific Technology Ltd., Tokyo) operating at constant flow rate 0.6 m^3^/min. Samples were continuously collected for 1 week, after which the QFFs were changed during the sampling period. After atmospheric sampling, QFF weights were determined and cut out for analysis of ClPAHs, PAHs, ions, elements, and carbons. The QFFs were wrapped in aluminum foil and sealed, and stored in a freezer at −35 °C until extraction.

### ClPAH and PAH analysis

After collection of atmospheric samples, extraction and cleanup of ClPAHs and PAHs associated with particles were extracted by a previously reported method^33^. Chemical analyses were done with a JMS-Q1000GC quadrupole MS (JEOL, Tokyo) equipped with a 7890 A (Agilent Technologies, CA) GC with an InertCap 5MS/NP capillary column (30 m × 0.25 mm i.d. × 0.25 μm film thickness, GL Science Inc., Tokyo). The MS was operated in selected ion monitoring mode. The ion current was held at 200 μA. Helium was used as a carrier gas at flow rate 1.0 mL/min. In ClPAH analysis, oven temperature was kept at 100 °C for 2 min, then increased from 100 to 200 °C at a rate of 25 °C/min until the oven reached 200 °C with no hold, and then increased from 200 to 300 °C at a rate of 5 °C/min, then kept at 300 °C for 15 min. Temperatures of the injector and GC-MS transfer line were maintained at 300 °C and 280 °C, respectively. The MS system was run in the electron impact ionization mode, and electron energy was 70 eV. Detailed information of GC-MS analytical conditions are described elsewhere^33^.

Analytical recoveries of the targeted 24 ClPAHs in the QFF samples (n = 6) ranged from 93.2 ± 4% (Cl_2_BaP) to 106.4 ± 2% (1-ClPy), indicating that the analytical method was appropriate for ClPAH analysis. Similar quantities were obtained from analysis of 22 PAHs, from 62.3 ± 1% (DBahP) through 114.1 ± 1% (BaA). Furthermore, recoveries of the internal standard spiked into individual samples were 95 ± 6% for Fluor-d_10_ and 97 ± 6% for Pery-d_12_. None of the targeted ClPAHs was detected in procedural blank samples. The limit of detection of ClPAHs was estimated by three times the standard deviation from repeated (n = 6) analyses of a diluted standard solution, and ranged from 0.01 (9-ClPhe) to 0.55 pg/m^3^ (Cl_3_BaP) based on air volumes of 6,048 m^3^ on the QFFs.

### Ion analysis

All QFF portions collected were used for identification of water-soluble inorganic anions and cations. For extraction of inorganic ions, both sample and blank QFFs were extracted in an ultrasonic bath for 20 min with 10 ml deionized water. After passing through microporous membranes (0.2 μm pore size, Ekicrodisc 13, Wako Pure Chemicals), ionic concentrations in aqueous extracts were determined by ion chromatography (ICS-1000, DIONEX, Tokyo) with a conductivity detector. For anions, the analytical column used was IonPac AS9-HC (DIONEX) at 40 °C. A 9-mM sodium carbonate aqueous solution was used as eluent, at flow rate 1.0 mL/min. The analytical column used for cations was IonPac CS12A (DIONEX) at 40 °C. A 20-mM methanesulfonic acid aqueous solution was used as eluent, at flow rate 1.0 mL/min.

### Carbon analysis

All QFF portions were analyzed using a carbon analyzer (Sunset Laboratory Inc., Tigard, Oregon, USA) with interagency monitoring of protected visual environments (IMPROVE) as the thermal/optical method. The analytical temperature was increased by IMPROVE, which heated the sample to 120, 250, 450, and 550 °C in a pure He atmosphere to determine OC1–OC4, and subsequently to 550, 700, 800 °C in a 2% O_2_/98% He atmosphere to determine EC1–EC3. Carbon was defined as pyOC by repartitioning. OC is defined as OC1 + OC2 + OC3 + OC4 + pyOC and EC as EC1 + EC2 + EC3.

### Element analysis

Twelve species of elements were targeted as follows, with abbreviations: sulfur (S), potassium (K), calcium (Ca), titanium (Ti), vanadium (V), chromium (Cr), manganese (Mn), iron (Fe), nickel (Ni), copper (Cu), zinc (Zn), and lead (Pb). These elements were determined using energy dispersive X-ray fluorescence spectrometry (EDXRF, Rigaku Corp., Tokyo) coupled with fundamental parameter quantification. Analytical conditions and quality controls were confirmed and are reported elsewhere^34^.

### PMF

PMF is a multivariable receptor model that is widely used for source apportionment studies of particulate matter. The PMF model basically assumes that the number of factors is *p*, i.e., several sources impacting a receptor, and that the linear combinations of these impacts from the *p* factors give rise to the observed concentrations of various species^35^. The model can be expressed as


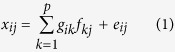


here, *x*_*ij*_ is the observed concentration for the *j*^*th*^ species in the *i*^*th*^ sample, *g*_*ik*_ is the contribution of the *k*^*th*^ factor to the observed concentration of the *i*^*th*^ sample, *f*_*kj*_ is the chemical profile of the *k*^*th*^ factor that is the *j*^*th*^ species, and *e*_*ij*_ is the residual for the *j*^*th*^ species in the *i*^*th*^ sample. The requirement of PMF is to minimize the objective function *Q*(*E*), expressed as


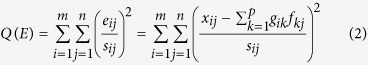


here, *s*_*ij*_ is the uncertainty in the *j*^*th*^ element for the *i*^*th*^ sample, with the constraint that all contributions and profiles be non-negative.

We used EPA PMF 5.0 to identify possible sources of TSP at the industrial and residential sites^36^. The dataset used in the PMF analysis combined each dataset of industrial and residential site. The concentration matrix consisted of carbons, water-soluble ions, elements, and major PAHs and ClPAHs. Observations from both sites were merged into one matrix, and data less than the method detection limit (MDL) were replaced by 1/2 MDL. Uncertainties of the data were estimated using the following equations^37^.


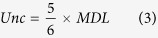



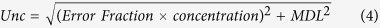


In case the concentration was less than the MDL, the uncertainty was calculated by Eq. (3). If the concentration was greater than the MDL, the uncertainty was calculated by Eq. (4), in which the error fraction was assumed to be 15%. In addition, extra modeling uncertainty (5%) was imposed to consider the variability of source profiles between the two monitoring sites. The categorization of TSP was set to “weak” to avoid strong influence on the solution. Because several organic species such as oxalate ion, PAHs, and ClPAHs were included in the input dataset with OC, the categorization of OC was also set to weak for the same reason as TSP. All other species having signal to noise ratio >2 were categorized as “strong”. To determine the optimal number of factors, a base run was tested for 6–10 factors with a fixed seed, verifying *Q* value stability, the *Q*/*Qexp* of samples and species, *r*^2^, scaled residuals, resulting source profiles and contributions. Furthermore, bootstrap and Fpeak runs were conducted to estimate uncertainty in the base run solution.

## Additional Information

**How to cite this article:** Kamiya, Y. *et al*. Source apportionment of chlorinated polycyclic aromatic hydrocarbons associated with ambient particles in a Japanese megacity. *Sci. Rep.* **6**, 38358; doi: 10.1038/srep38358 (2016).

**Publisher's note:** Springer Nature remains neutral with regard to jurisdictional claims in published maps and institutional affiliations.

## Supplementary Material

Supplementary Information
