# Supplementary material for: Source apportionment of chlorinated polycyclic aromatic hydrocarbons associated with ambient particles in a Japanese megacity
Source: Sci Rep. 2016 Dec 6;6:38358. doi: 10.1038/srep38358 (PMC5138597; doi:10.1038/srep38358)
Supplement: Supplementary Information [file srep38358-s1.pdf]

Supplementary Information

Source apportionment of chlorinated aromatic hydrocarbons associated with ambient particles in a Japanese megacity

Yuta Kamiya[1], Akihiro Iijima[2], Fumikazu Ikemori [3], Tomoaki Okuda[4] and Takeshi Ohura[1,*]

[1] Graduate School of Agriculture, Meijo University, 1-501 Shiogamaguchi, Nagoya 468-8502, Japan

[2] Nagoya City Institute for Environmental Science, 5-16-8 Toyoda, Nagoya 457-0841, Japan

[3] Faculty of Regional Policy, Takasaki City University of Economics, 1300 Kaminamie, Takasaki 370-0801, Japan

[4] Department of Applied Chemistry, Faculty of Science and Technology, Keio University, 3-14-1 Hiyoshi, Kohoku-ku, Yokohama 223-8522, Japan

* Corresponding author:

E-mail: ohura@meijo-u.ac.jp, Phone: +81-52-838-2438; Fax: +81-52-833-5524

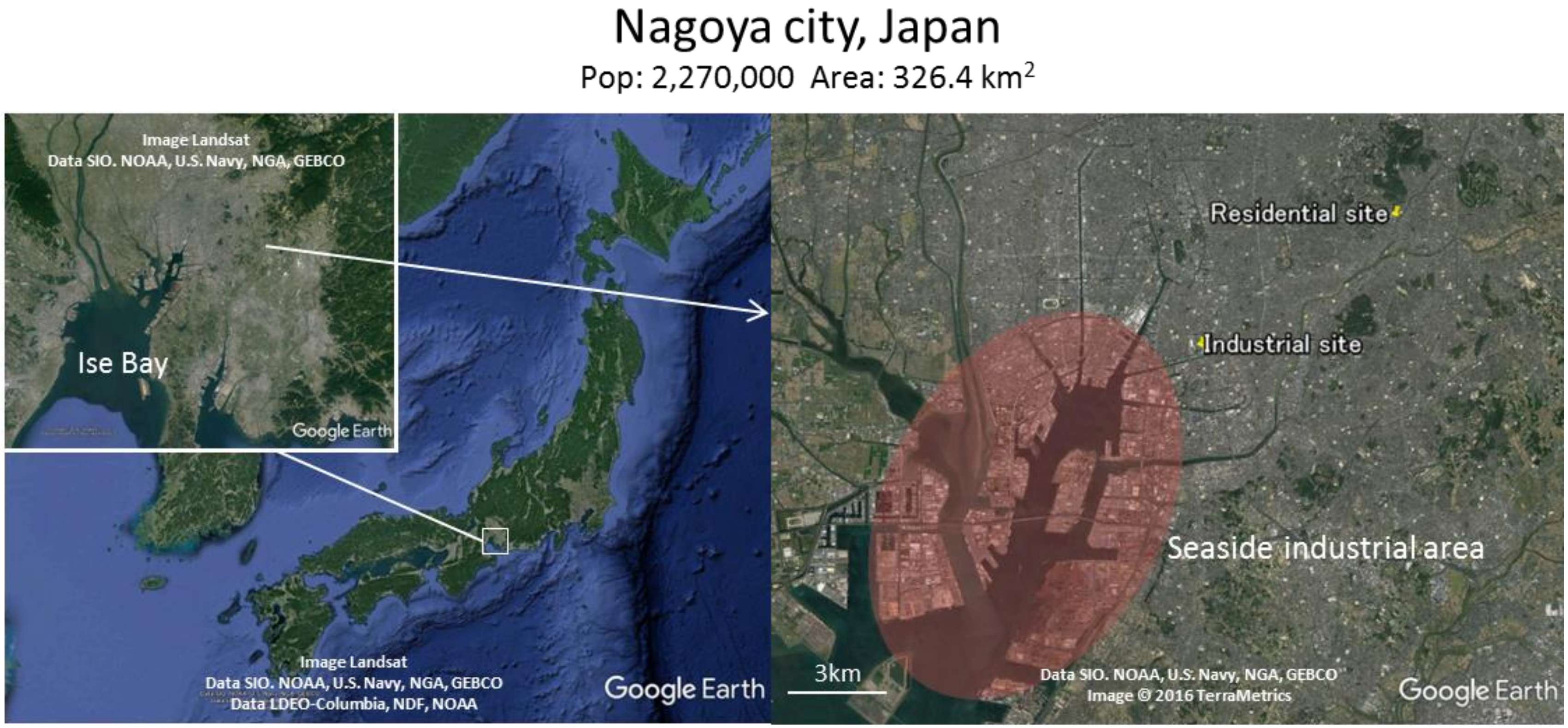

Figure S1 Google Earth (ver. 7.1.7) images of sampling sites in Nagoya city, Japan. These images were illustrated by Microsoft PowerPoint 2010

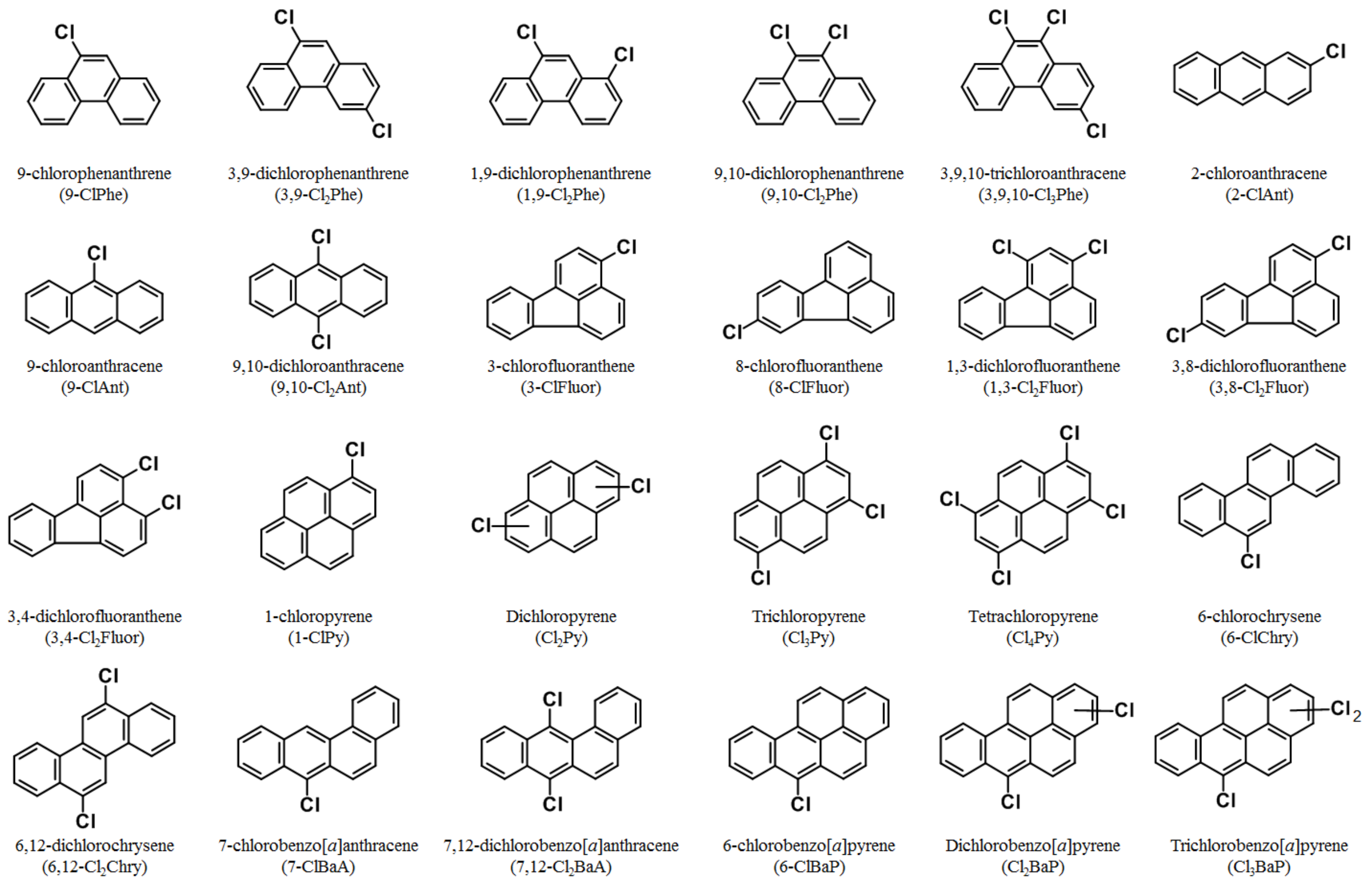

Figure S2 Structures of 24 ClPAHs targeted in this study

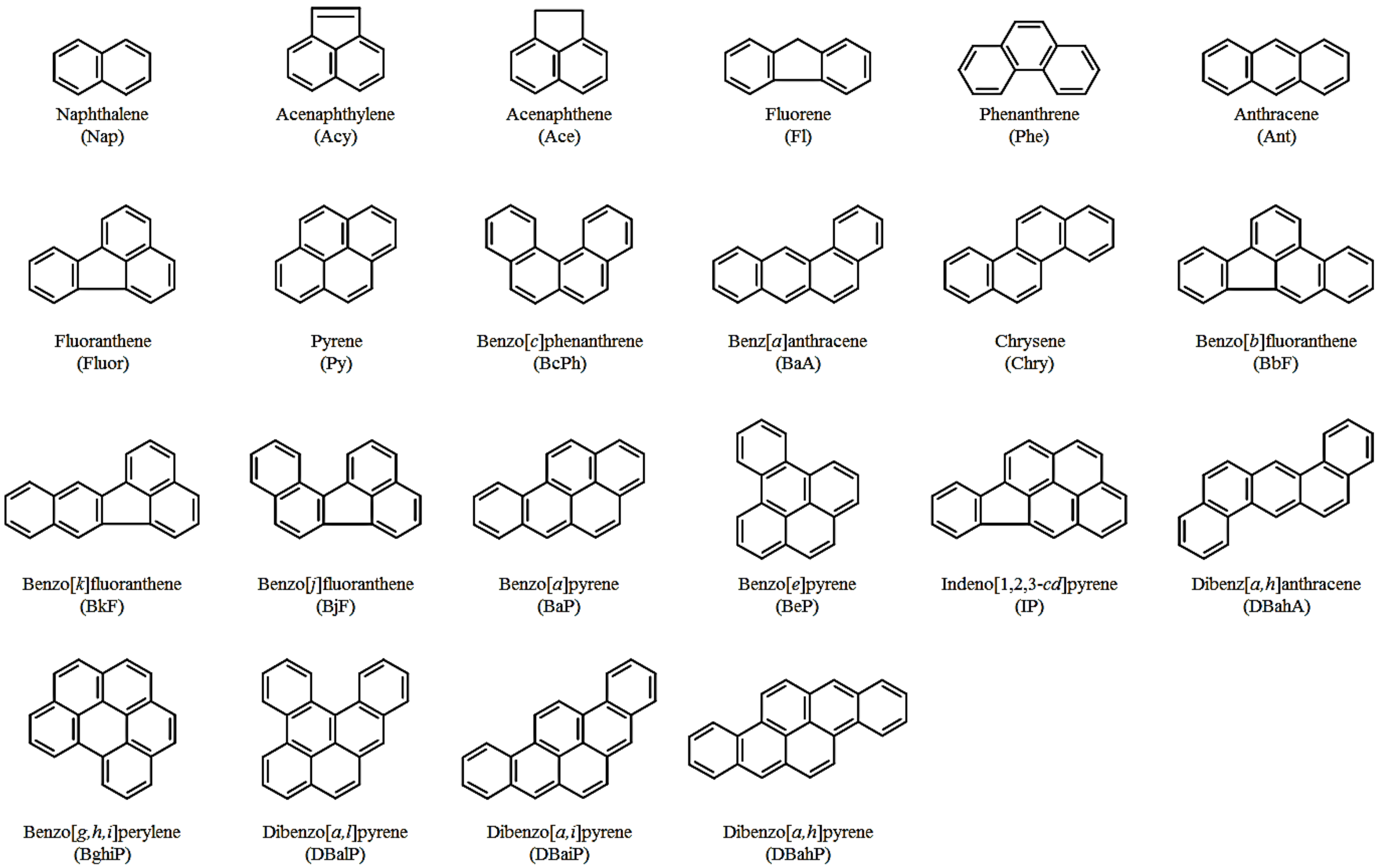

Figure S3 Structures of 22 PAHs targeted in this study

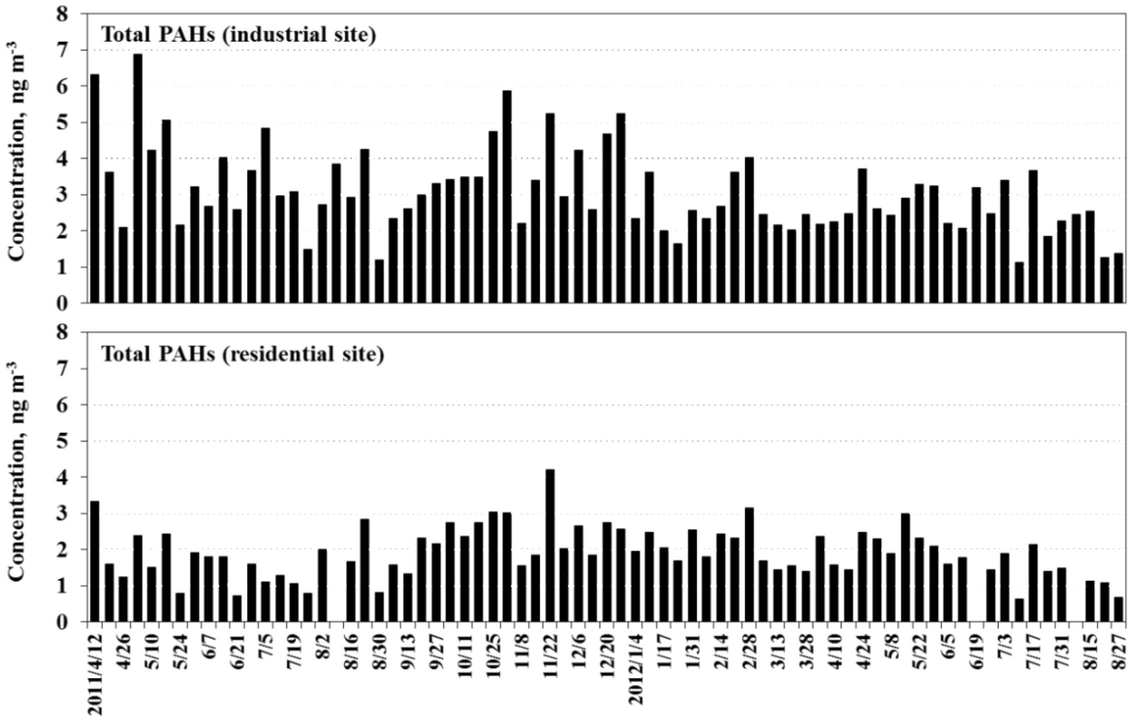

Figure S4 Weekly variation of total PAH concentrations in industrial (upper) and residential site (bottom)

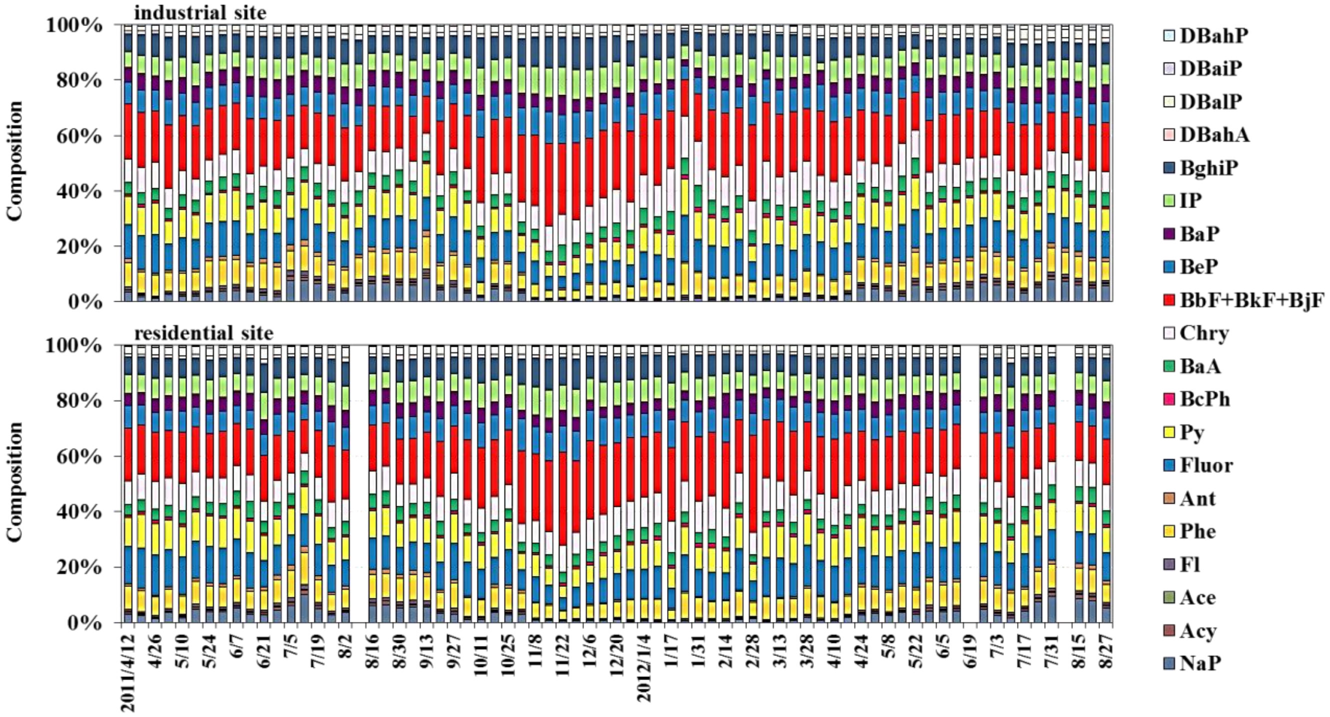

Figure S5 Weekly variation of composition of total PAHs in TSP at industrial (upper) and residential site (bottom)

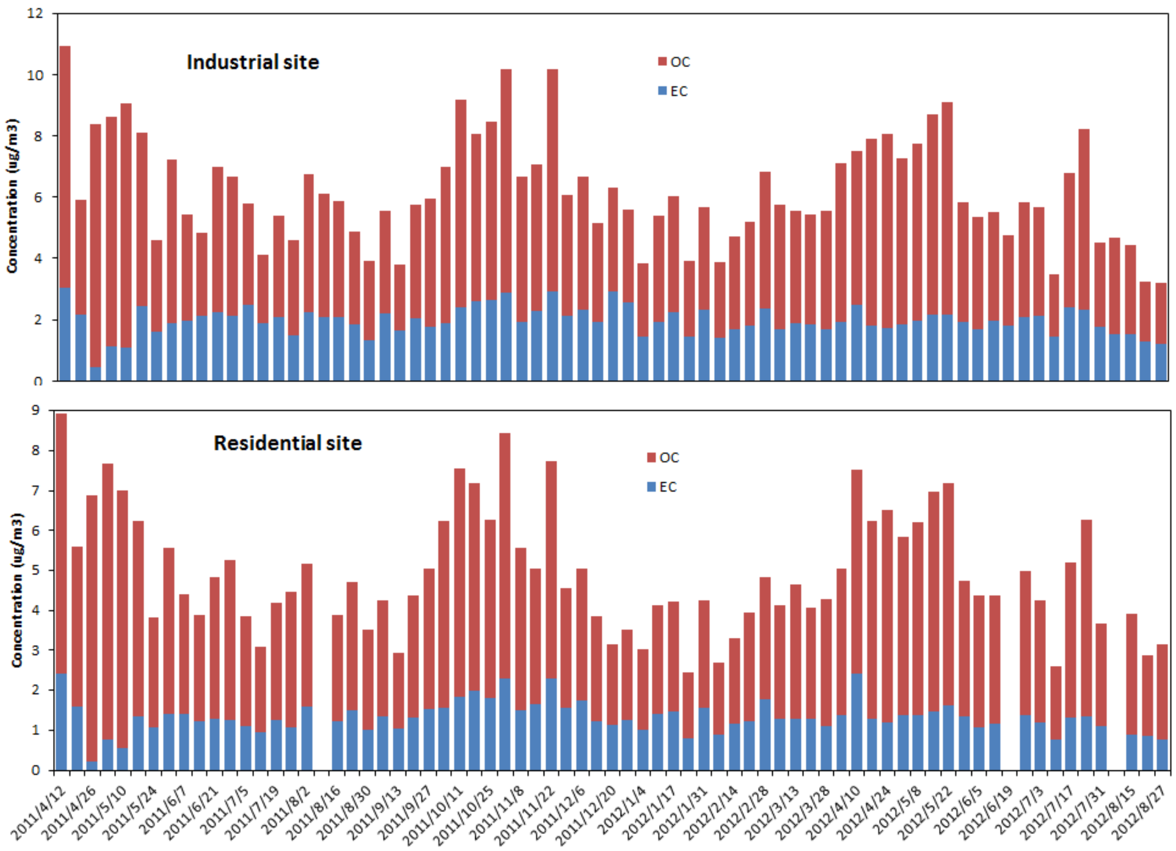

Figure S6 Weekly variation of OC and EC concentrations in TSP at industrial (upper) and residential site (bottom)

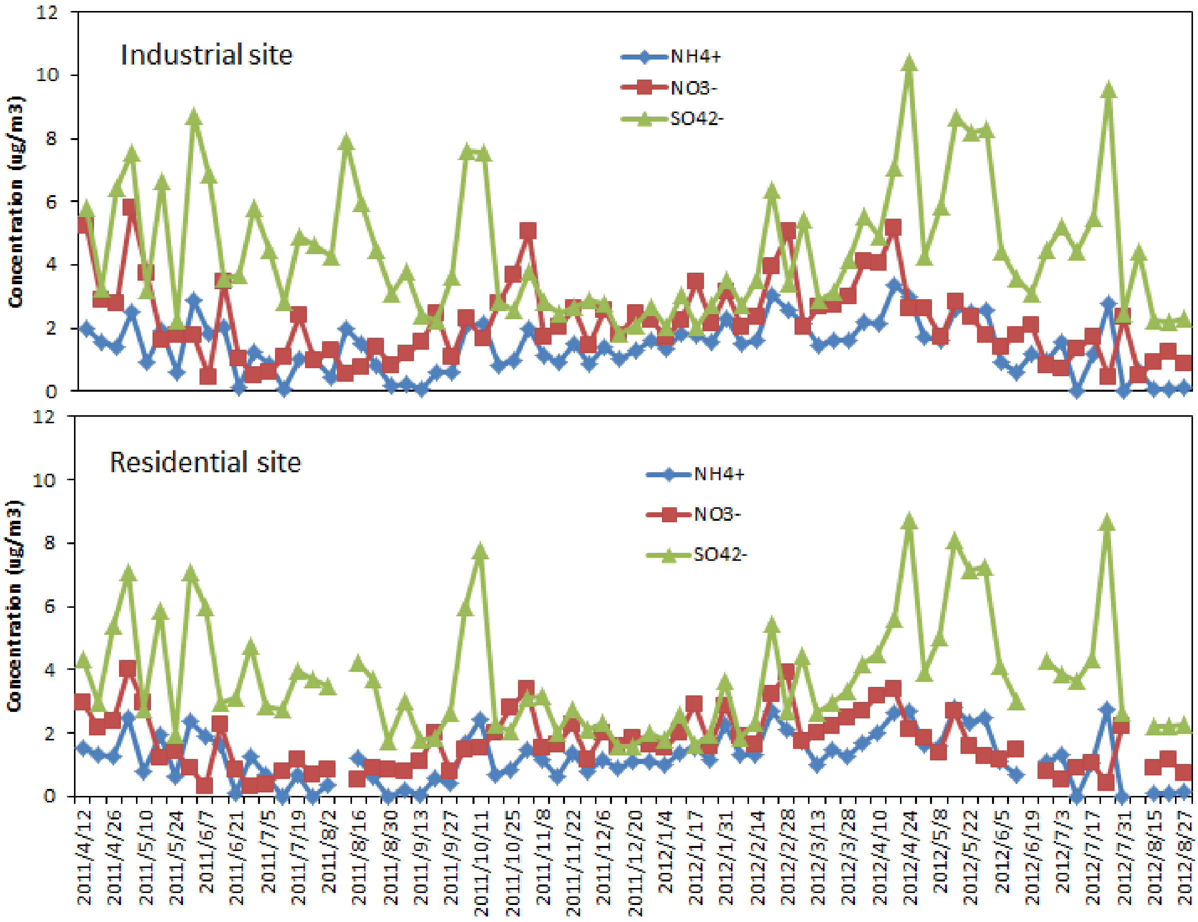

Figure S7 Weekly variation of NH_4^+, NO_3^+ and SO_4^{2-} concentrations in TSP at industrial (upper) and residential site (bottom)

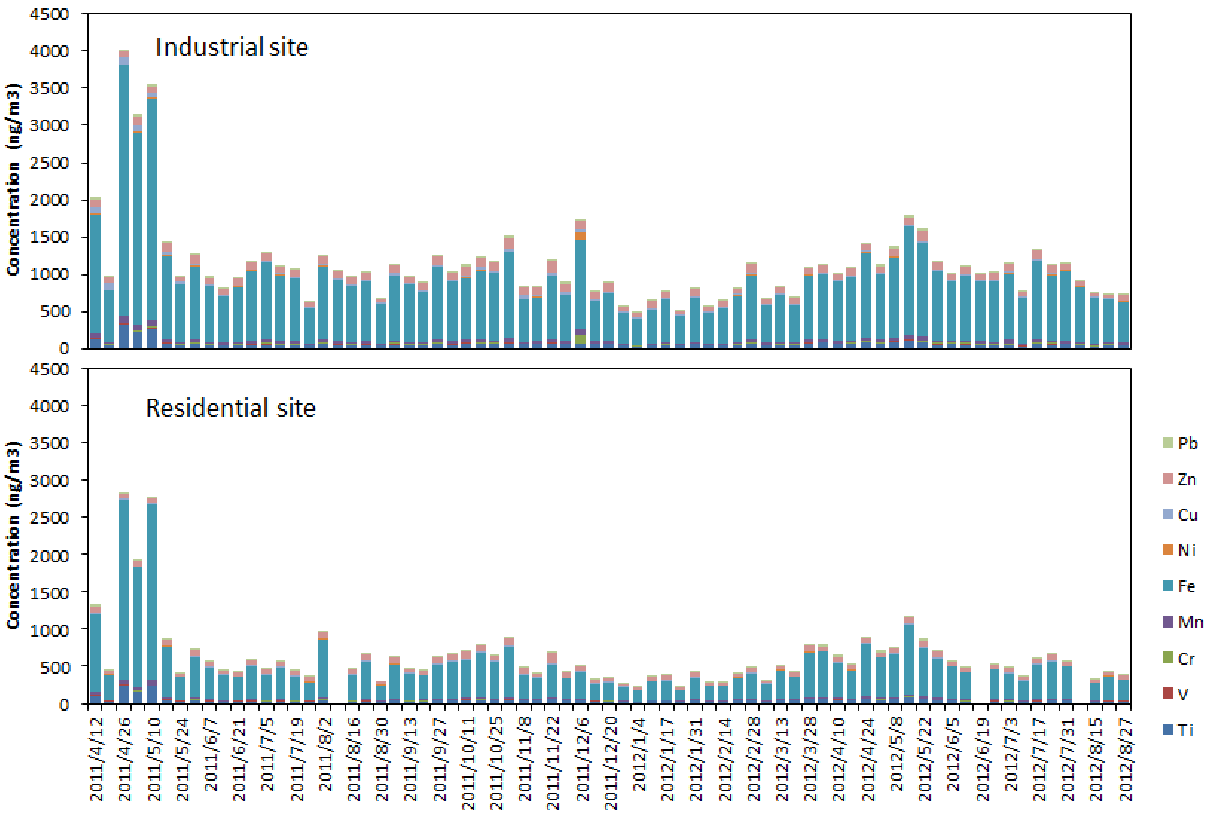

Figure S8 Weekly variation of element concentrations in TSP at industrial (upper) and residential site (bottom)

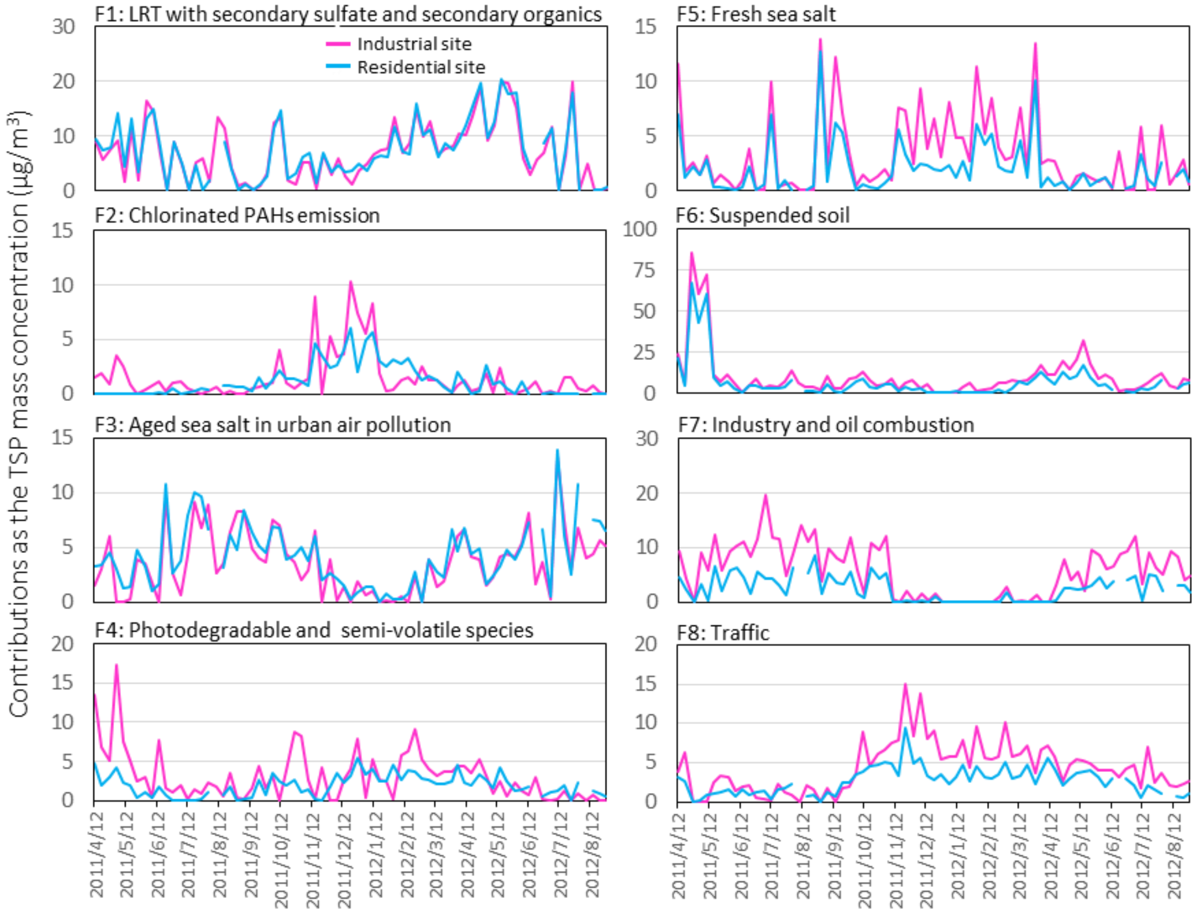

Figure S9 The weekly contributions of TSP for the eight sources identified by PMF

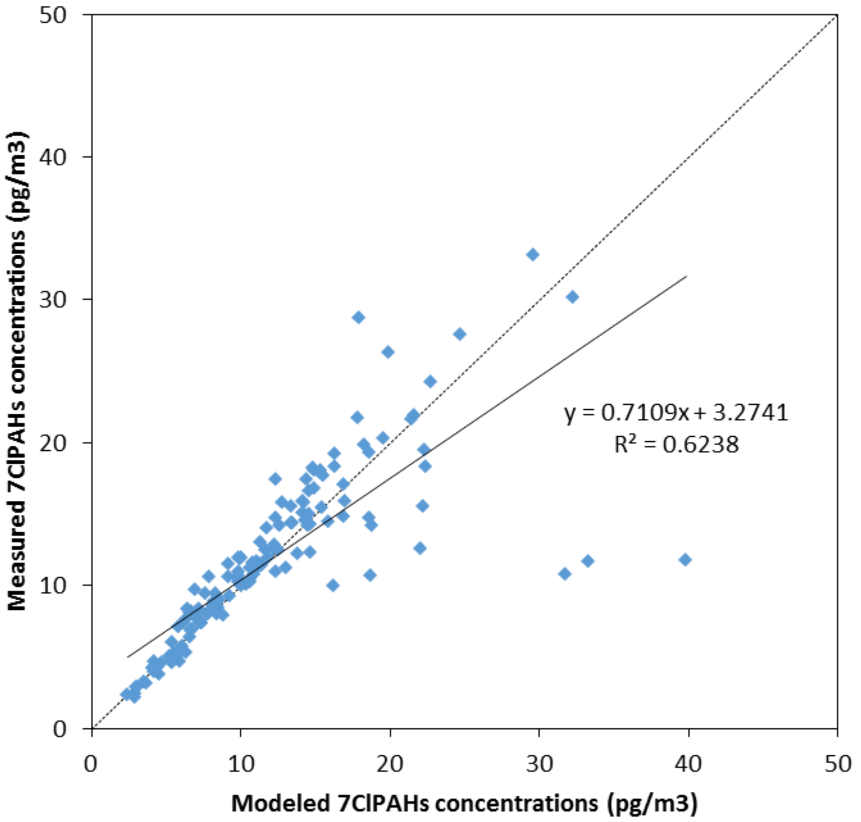

Figure S10 The relationship of concentrations between modeled and measured 7 ClPAHs used in PMF analysis

Table S1 Concentrations of ClPAHs in TSP at industrial and residential site in Nagoya, Japan

Compound	Industrial site				Residential site				I/R
	Mean	SD	min	max	Mean	SD	min	max	
ClPAH (pg/m^3)									
9-ClPhe	0.53	0.26	0.20	1.98	0.40	0.22	<0.01	1.79	1.3
2-ClAnt	0.42	0.12	0.17	0.81	0.42	0.12	0.16	0.73	1.0
9-ClAnt	0.20	0.12	0.02	0.68	0.11	0.09	<0.01	0.31	1.9
3,9-Cl_2Phe	0.01	0.03	<0.02	0.12	0.03	0.05	<0.02	0.12	0.3
9,10-Cl_2Ant +1,9-Cl_2Phe	0.14	0.11	<0.02	0.63	0.14	0.06	0.04	0.26	1.0
9,10-Cl_2Phe	0.12	0.08	0.05	0.65	0.12	0.05	<0.02	0.32	1.0
3-ClFluor	0.63	0.35	0.09	1.50	0.51	0.30	0.05	1.27	1.2
8-ClFluor	0.15	0.09	0.02	0.48	0.14	0.09	<0.01	0.53	1.1
1-ClPy	3.02	1.33	0.76	7.35	1.97	0.89	0.49	3.88	1.5
3,9,10-Cl_3Phe	0.23	0.29	<0.03	2.13	0.21	0.11	<0.03	0.46	1.1
1,3-Cl_2Fluor	0.04	0.06	<0.03	0.20	0.10	0.13	<0.03	0.44	0.4
3,8-Cl_2Fluor	0.27	0.25	0.08	1.77	0.21	0.13	<0.03	0.55	1.3
Cl_2Py	0.53	0.27	0.06	1.33	0.43	0.18	0.19	0.81	1.2
3,4-Cl_2Fluor	0.31	0.20	<0.04	1.08	0.27	0.12	0.09	0.91	1.2
6-ClChry	0.81	0.68	<0.01	4.47	0.35	0.16	<0.01	0.72	2.3
7-ClBaA	2.12	1.30	0.56	6.22	1.11	0.49	0.39	2.42	1.9
Cl_3Py	0.55	0.57	<0.07	3.97	0.50	0.22	<0.07	0.98	1.1
6,12-Cl_2Chry	0.05	0.05	<0.04	0.20	0.17	0.08	<0.04	0.40	0.3
7,12-Cl_2BaA	0.12	0.08	<0.04	0.38	0.09	0.09	<0.04	0.24	1.2
Cl_4Py	0.43	0.40	0.10	1.85	0.45	0.23	<0.04	1.15	1.0
6-ClBaP	7.16	7.11	1.26	45.95	4.38	3.08	0.67	15.69	1.6
Cl_2BaP	2.13	4.14	0.20	32.35	1.50	1.62	0.14	9.49	1.4
Cl_3BaP	0.69	0.92	<0.45	7.03	0.46	0.46	<0.45	2.29	1.5
Total ClPAHs	20.67	14.61	5.67	104.78	14.10	7.02	3.42	33.68	1.5

I/R: Ratio of industrial site/residential site, < shows below the limit of quantitation

Table S2 Concentrations of PAHs in TSP at industrial and residential site in Nagoya, Japan

Compound	Industrial site				Residential site				I/R
	Mean	SD	min	max	Mean	SD	min	max	
PAH (ng m^{-3})									
Nap	0.102	0.076	0.013	0.368	0.053	0.0392	0.007	0.186	1.9
Acy	0.021	0.013	0.005	0.072	0.012	0.0062	0.003	0.033	1.8
Ace	0.005	0.003	0.001	0.014	0.003	0.0012	0.001	0.006	1.5
Fl	0.026	0.016	0.006	0.091	0.013	0.0066	0.005	0.033	1.9
Phe	0.217	0.087	0.084	0.550	0.136	0.0495	0.044	0.281	1.6
Ant	0.031	0.020	0.007	0.107	0.016	0.0087	0.005	0.046	1.9
Fluor	0.320	0.121	0.105	0.790	0.215	0.0790	0.061	0.445	1.5
Py	0.269	0.098	0.092	0.640	0.176	0.0601	0.052	0.344	1.5
BcPh	0.029	0.011	0.010	0.062	0.020	0.0078	0.006	0.049	1.4
BaA	0.146	0.055	0.055	0.322	0.082	0.0283	0.030	0.167	1.8
Chry	0.276	0.108	0.088	0.567	0.178	0.0690	0.054	0.402	1.5
BbF+BkF+BjF	0.615	0.300	0.182	1.580	0.392	0.2207	0.113	1.415	1.6
BeP	0.252	0.117	0.079	0.640	0.157	0.0703	0.050	0.393	1.6
BaP	0.166	0.078	0.039	0.458	0.097	0.0409	0.036	0.243	1.7
IP	0.232	0.112	0.064	0.571	0.143	0.0604	0.051	0.377	1.6
DBahA	0.247	0.118	0.085	0.582	0.144	0.0629	0.055	0.414	1.7
BghiP	0.043	0.021	0.011	0.114	0.023	0.0096	0.009	0.060	1.9
DBalP	0.073	0.032	0.019	0.172	0.049	0.0190	0.018	0.113	1.5
DBaiP	0.015	0.008	0.003	0.050	0.007	0.0027	0.003	0.013	1.9
DBahP	0.004	0.003	ND	0.014	0.003	0.0008	0.001	0.005	1.5
Parent-PAHs	1.425	0.504	0.495	3.211	0.902	0.301	0.287	1.650	1.6
Total PAHs	3.093	1.174	1.123	6.888	1.923	0.7008	0.624	4.197	1.6

I/R: Ratio of Industrial site/Urban site, < shows below the limit of quantitation

Table S3 Concentrations of carbons in TSP at industrial and residential site in Nagoya, Japan

	Industry site				Residential site				
	Mean	SD	min	max	Mean	SD	min	max	I/R
Carbon (ug/m3)									
OC1	0.05	0.05	0.00	0.22	0.03	0.03	0.00	0.16	1.3
OC2	1.05	0.37	0.37	2.12	0.90	0.32	0.33	1.71	1.2
OC3	1.34	0.46	0.65	2.77	1.06	0.43	0.39	2.30	1.3
OC4	0.34	0.09	0.22	0.70	0.32	0.09	0.18	0.58	1.1
pyOC	1.49	0.88	0.52	4.18	1.30	0.73	0.47	3.55	1.1
EC1	1.60	0.49	0.00	2.71	1.07	0.40	0.00	2.16	1.5
EC2	0.30	0.15	0.09	0.71	0.21	0.09	0.08	0.52	1.4
EC3	0.08	0.03	0.02	0.14	0.05	0.02	0.01	0.10	1.6
OC	4.25	1.57	1.97	7.98	3.61	1.32	1.65	6.89	1.2
EC	1.97	0.46	0.46	3.02	1.33	0.40	0.22	2.42	1.5

I/R: Ratio of Indstrial site/Residential site

Table S4 Concentrations of water-soluble ions in TSP at industrial and residential site in Nagoya, Japan

	Industry site				Residential site				
	Mean	SD	min	max	Mean	SD	min	max	I/R
Ion (ug/m3)									
Na^+	0.94	0.44	0.30	2.43	0.80	0.40	0.21	2.10	1.2
$NH4^+$	1.39	0.83	0.02	3.34	1.23	0.79	0.00	2.80	1.1
K^+	0.18	0.05	0.09	0.34	0.14	0.04	0.07	0.25	1.2
Mg^{2+}	0.15	0.07	0.04	0.32	0.10	0.05	0.02	0.22	1.5
Ca^{2+}	0.70	0.33	0.30	1.92	0.44	0.28	0.16	1.43	1.6
Cl^-	0.43	0.46	0.01	2.62	0.26	0.25	0.00	1.16	1.7
NO_3^-	2.18	1.25	0.45	5.79	1.70	0.90	0.33	4.02	1.3
SO_4^{2-}	4.45	2.09	1.81	10.40	3.74	1.85	1.59	8.72	1.2
$(COO^-)_2$	0.31	0.14	0.03	0.56	0.27	0.12	0.07	0.58	1.1
Cation	0.17	0.05	0.07	0.30	0.14	0.05	0.07	0.26	1.2
Anion	0.14	0.05	0.06	0.26	0.11	0.04	0.06	0.22	1.2

I/R: Ratio of Indstrial site/Residential site

Table S5 Concentrations of elements in TSP at industrial and residential site in Nagoya, Japan

	Industry site				Residential site				
	Mean	SD	min	max	Mean	SD	min	max	I/R
Metal (ng/m3)									
S	1245.49	465.06	545.87	2636.02	867.93	352.36	99.08	2014.59	1.4
K	274.34	229.83	101.49	1466.22	196.82	181.24	72.46	1090.15	1.4
Ca	755.79	361.22	386.13	2306.59	467.16	295.18	195.27	1733.95	1.6
Ti	65.02	47.13	30.03	327.17	42.76	39.01	17.69	242.41	1.5
V	5.39	2.98	0.54	11.99	3.45	2.07	0.54	10.31	1.6
Cr	12.19	13.87	3.85	125.21	5.31	2.02	1.66	10.86	2.3
Mn	34.83	13.81	15.97	88.57	18.15	10.57	5.30	64.79	1.9
Fe	880.27	503.30	348.82	3379.12	482.29	398.95	147.40	2416.37	1.8
Ni	7.72	10.44	1.80	93.37	3.50	1.25	0.94	6.07	2.2
Cu	25.43	17.69	11.35	91.33	10.06	2.74	5.31	21.76	2.5
Zn	91.41	21.82	46.29	145.19	61.49	17.66	29.47	126.39	1.5
Pb	20.39	7.00	6.93	37.98	17.60	5.63	0.00	31.36	1.2

I/R: Ratio of Indstrial site/Residential site

Table S6 Relationship among individual PAH concentrations in the industrial site

Test of no correlation [upper triangle: P value/lower triangle: judgment (*: 5%, **: 1%)]

	1	2	3	4	5	6	7	8	9	10	11	12	13	14	15	16	17	18	19	20
1 Nap	-	0.00	0.00	0.00	0.00	0.00	0.00	0.00	0.71	0.00	0.91	0.49	0.15	0.00	0.25	0.34	0.00	0.00	0.00	0.07
2 Acy	**	-	0.00	0.00	0.00	0.00	0.00	0.00	0.12	0.00	0.17	0.10	0.01	0.00	0.06	0.09	0.00	0.00	0.00	0.04
3 Ace	**	**	-	0.00	0.00	0.00	0.00	0.00	0.28	0.00	0.38	0.14	0.02	0.00	0.06	0.08	0.00	0.00	0.00	0.28
4 Fl	**	**	**	-	0.00	0.00	0.00	0.00	0.02	0.00	0.06	0.02	0.00	0.00	0.01	0.02	0.00	0.00	0.00	0.99
5 Phe	**	**	**	**	-	0.00	0.00	0.00	0.00	0.00	0.00	0.00	0.00	0.00	0.00	0.00	0.00	0.00	0.00	0.24
6 Ant	**	**	**	**	**	-	0.00	0.00	0.10	0.00	0.17	0.05	0.01	0.00	0.03	0.03	0.00	0.00	0.00	0.51
7 Fluor	**	**	**	**	**	**	-	0.00	0.00	0.00	0.00	0.00	0.00	0.00	0.00	0.00	0.00	0.00	0.01	0.09
8 Py	**	**	**	**	**	**	**	-	0.00	0.00	0.00	0.00	0.00	0.00	0.00	0.00	0.00	0.00	0.00	0.12
9 BcPh				*	**		**	**	-	0.00	0.00	0.00	0.00	0.00	0.00	0.00	0.00	0.00	0.31	0.15
10 BaA	**	**	**	**	**	**	**	**	**	-	0.00	0.00	0.00	0.00	0.00	0.00	0.00	0.00	0.01	0.65
11 Chry					**		**	**	**	**	-	0.00	0.00	0.00	0.00	0.00	0.00	0.00	0.35	0.12
12 BbF+BkF+BjF				*	**		**	**	**	**	**	-	0.00	0.00	0.00	0.00	0.00	0.00	0.15	0.12
13 BeP		*	*	**	**	**	**	**	**	**	**	**	-	0.00	0.00	0.00	0.00	0.00	0.05	0.17
14 BaP	**	**	**	**	**	**	**	**	**	**	**	**	**	-	0.00	0.00	0.00	0.00	0.00	0.61
15 IP				*	**	*	**	**	**	**	**	**	**	**	-	0.00	0.00	0.00	0.04	0.50
16 BghiP				*	**	*	**	**	**	**	**	**	**	**	**	-	0.00	0.00	0.10	0.26
17 DBahA	**	**	**	**	**	**	**	**	**	**	**	**	**	**	**	**	-	0.00	0.00	0.81
18 DBalP	**	**	**	**	**	**	**	**	**	**	**	**	**	**	**	**	**	-	0.00	0.17
19 DBaiP	**	**	**	**	**	**	**	**		**			*	**	*		**	**	-	0.00
20 DBahP		*																	**	-

Table S7 Relationship among individual ClPAH concentrations in the industrial site

Test of no correlation [upper triangle: P value/lower triangle: judgment (*: 5%, **: 1%)]

	1	2	3	4	5	6	7	8	9	10	11	12	13	14	15	16	17	18	19	20	21	22	23
1 9-ClPhe	-	0.01	0.02	0.36	0.05	0.09	0.00	0.06	0.00	0.12	0.23	0.10	0.02	0.13	0.01	0.03	0.10	0.23	0.52	0.00	0.07	0.19	0.16
2 2-ClAnt	*	-	0.02	0.59	0.50	0.00	0.00	0.00	0.00	0.00	0.51	0.00	0.00	0.26	0.12	0.00	0.54	0.33	0.78	0.12	0.01	0.11	0.17
3 9-ClAnt	*	*	-	0.19	0.55	0.34	0.55	0.32	0.25	0.12	0.13	0.06	0.63	0.00	0.00	0.73	0.28	0.00	0.24	0.18	0.50	0.34	0.91
4 3,9-Cl_2Phe				-	0.02	0.44	0.88	0.06	0.65	0.99	0.26	0.01	0.39	0.08	0.79	0.34	0.02	0.16	0.02	0.42	0.57	0.92	0.43
5 9,10-Cl_2Ant+1,9-Cl_2Phe	*			*	-	0.46	0.01	0.14	0.04	0.44	0.25	0.01	0.38	0.15	0.48	0.61	0.06	0.16	0.38	0.51	0.93	0.91	0.73
6 9,10-Cl_2Phe		**				-	0.00	0.01	0.00	0.00	0.01	0.00	0.00	0.48	0.55	0.00	0.00	0.03	0.20	0.02	0.01	0.02	0.11
7 3-ClFluor	**	**			*	**	-	0.00	0.00	0.00	0.00	0.00	0.00	0.84	0.01	0.00	0.13	0.00	0.26	0.03	0.00	0.00	0.00
8 8-ClFluor		**				**	**	-	0.00	0.03	0.13	0.00	0.06	0.00	0.52	0.01	0.17	0.16	0.39	0.42	0.00	0.01	0.04
9 1-ClPy	**	**			*	**	**	**	-	0.00	0.00	0.00	0.00	0.90	0.02	0.00	0.19	0.01	0.72	0.08	0.00	0.00	0.00
10 3,9,10-Cl_3Phe		**				**	**	*	**	-	0.00	0.00	0.00	0.44	0.55	0.00	0.00	0.03	0.42	0.01	0.00	0.01	0.03
11 1,3-Cl_2Fluor						*	**		**	**	-	0.01	0.08	0.08	0.25	0.17	0.62	0.00	0.57	0.39	0.02	0.15	0.12
12 3,8-Cl_2Fluor		**		*	**	**	**	**	**	**	**	-	0.00	0.61	0.07	0.00	0.87	0.01	0.95	0.00	0.00	0.00	0.00
13 Cl_2Py	*	**				**	**		**	**		**	-	0.03	0.00	0.00	0.00	0.00	0.12	0.00	0.00	0.00	0.00
14 3,4-Cl_2Fluor			**					**					*	-	0.00	0.92	0.00	0.16	0.30	0.75	0.57	0.86	0.25
15 6-ClChry	**		**				**		*				**	**	-	0.04	0.03	0.18	0.74	0.24	0.10	0.83	0.42
16 7-ClBaA	*	**				**	**	**	**	**		**	**		*	-	0.15	0.00	0.38	0.00	0.00	0.00	0.00
17 Cl_3Py				*		**				**			**	**	*		-	0.46	0.01	0.00	0.09	0.11	0.06
18 6,12-Cl_2Chry			**			*	**		**	*	**	**	**			**		-	0.78	0.03	0.08	0.45	0.77
19 7,12-Cl_2BaA				*													**		-	0.85	0.34	0.10	0.13
20 Cl_4Py	**					*	*			**		**	**			**	**	*		-	0.01	0.05	0.12
21 6-ClBaP		*				*	**	**	**	**	*	**	**			**				*	-	0.00	0.00
22 Cl_2BaP						*	**	**	**	**		**	**			**				*	**	-	0.00
23 Cl_3BaP							**	*	**	*		**	**			**					**	**	-

Table S8 Input data of PMF analysis

Species	Mean $\mu g/m^3$	Std. Dev. $\mu g/m^3$	% missing	% < MDL	S/N	Category
TSP	33.9	16.1	100%	0%	5.7	Weak
OC	3.94	1.48	100%	0%	5.7	Weak
EC	1.66	0.534	100%	0%	5.7	Strong
Na+	0.868	0.427	100%	0%	5.7	Strong
NH4+	1.31	0.814	100%	1%	5.5	Strong
Mg2+	0.129	0.0638	100%	0%	5.6	Strong
Cl-	0.343	0.380	100%	1%	5.5	Strong
NO3-	1.94	1.11	100%	0%	5.7	Strong
SO42-	4.10	2.00	100%	0%	5.7	Strong
(COO-)2	0.291	0.132	100%	0%	5.6	Strong
K	0.236	0.210	100%	0%	5.6	Strong
Ca	0.615	0.360	100%	0%	5.7	Strong
Ti	0.0541	0.0446	100%	0%	5.6	Strong
V	0.00444	0.00274	100%	5%	4.0	Strong
Cr	0.00882	0.0106	99%	0%	5.3	Strong
Mn	0.0267	0.0149	99%	0%	5.5	Strong
Fe	0.685	0.496	100%	0%	5.7	Strong
Ni	0.00565	0.00778	99%	0%	4.9	Strong
Cu	0.0179	0.0149	96%	0%	5.2	Strong
Zn	0.0768	0.0249	100%	0%	5.6	Strong
Pb	0.0190	0.00646	100%	1%	5.4	Strong
	ng/m^3	ng/m^3				
NaP	0.0779	0.0652	100%	0%	5.7	Strong
Phe	0.177	0.0814	100%	0%	5.7	Strong
Py	0.224	0.0939	100%	0%	5.7	Strong
BaA	0.115	0.0545	100%	0%	5.7	Strong
BaP	0.132	0.0715	100%	0%	5.7	Strong
IP	0.189	0.101	99%	0%	5.6	Strong
BghiP	0.197	0.108	100%	0%	5.7	Strong
9-ClPhe	0.000465	0.000248	99%	1%	5.6	Strong
1-ClPy	0.00251	0.00125	100%	0%	5.7	Strong
6-ClChry	0.000585	0.000546	100%	6%	5.3	Strong
7-ClBaA	0.00162	0.00111	100%	0%	5.7	Strong
6-ClBaP	0.00580	0.00567	100%	0%	5.7	Strong
Cl2BaP	0.00182	0.00317	99%	0%	5.6	Strong
Cl3BaP	0.000578	0.000738	100%	1%	5.3	Strong

Table S9 Summary of error estimation for TSP mass concentration.

Source		Unit	Base value	BS 5th	BS 50th	BS 95th	BS-DISP 5th	BS-DISP Ave.	BS-DISP 95th	DISP Min	DISP Ave.	DISP Max
LRT with secondary sulfate and secondary organics (Factor 1)	TSP	μg/m^3	7.4E+00	5.4E+00	7.0E+00	8.7E+00	5.0E+00	7.4E+00	9.7E+00	6.4E+00	8.0E+00	9.6E+00
Chlorinated PAHs emission (Factor 2)	TSP	μg/m^3	1.4E+00	8.1E-01	1.5E+00	2.5E+00	5.4E-01	1.8E+00	3.0E+00	8.0E-01	1.5E+00	2.1E+00
Aged sea salt in urban air pollution (Factor 3)	TSP	μg/m^3	3.8E+00	3.3E+00	4.2E+00	6.2E+00	2.9E+00	4.9E+00	6.9E+00	3.1E+00	4.9E+00	6.7E+00
Photodegradable and semi-volatile species (Factor 4)	TSP	μg/m^3	2.6E+00	9.2E-01	2.2E+00	4.9E+00	6.4E-01	2.9E+00	5.1E+00	1.7E+00	2.8E+00	4.0E+00
Fresh sea salt (Factor 5)	TSP	μg/m^3	2.4E+00	1.6E+00	2.5E+00	3.4E+00	1.2E+00	2.5E+00	3.8E+00	1.5E+00	2.2E+00	3.0E+00
Suspended soil (Factor 6)	TSP	μg/m^3	9.1E+00	6.7E+00	8.1E+00	9.2E+00	6.1E+00	8.3E+00	1.0E+01	6.6E+00	8.8E+00	1.1E+01
Industry and oil combustion (Factor 7)	TSP	μg/m^3	3.7E+00	1.4E+00	3.8E+00	6.5E+00	1.1E+00	4.2E+00	7.4E+00	1.7E+00	3.6E+00	5.5E+00
Traffic (Factor 8)	TSP	μg/m^3	3.1E+00	1.9E+00	3.9E+00	6.9E+00	1.4E+00	4.7E+00	8.0E+00	2.4E+00	3.8E+00	5.1E+00

Table S10 Summary of error estimation for specific elements as tracers of particular sources.

Source	Tracer	Unit	Base value	BS 5th	BS 50th	BS 95th	BS-DISP 5th	BS-DISP Ave.	BS-DISP 95th	DISP Min	DISP Ave.	DISP Max
LRT with secondary sulfate and secondary organics (Factor 1)	SO_4^{2-}	μg/m^3	2.1E+00	1.4E+00	1.9E+00	2.4E+00	1.4E+00	1.9E+00	2.5E+00	1.8E+00	2.1E+00	2.4E+00
Chlorinated PAHs emission (Factor 2)	6-ClBaP	ng/m^3	1.8E-03	1.1E-03	1.7E-03	2.4E-03	1.1E-03	1.9E-03	2.7E-03	1.4E-03	1.8E-03	2.2E-03
Aged sea salt in urban air pollution (Factor 3)	Na^+	μg/m^3	4.9E-01	3.9E-01	4.8E-01	5.9E-01	3.5E-01	4.9E-01	6.3E-01	4.0E-01	5.1E-01	6.2E-01
Photodegradable and semi-volatile species (Factor 4)	NO_3^-	μg/m^3	6.7E-01	1.1E-01	5.2E-01	8.9E-01	6.8E-02	5.1E-01	9.4E-01	5.0E-01	6.5E-01	8.1E-01
Fresh sea salt (Factor 5)	Cl^-	μg/m^3	2.7E-01	2.0E-01	2.5E-01	2.8E-01	2.0E-01	2.5E-01	3.0E-01	2.4E-01	2.8E-01	3.2E-01
Suspended soil (Factor 6)	Ca	μg/m^3	2.6E-01	1.8E-01	2.3E-01	2.8E-01	1.6E-01	2.4E-01	3.2E-01	1.8E-01	2.6E-01	3.4E-01
Industry and oil combustion (Factor 7)	V	μg/m^3	1.7E-03	1.1E-03	1.7E-03	2.1E-03	9.8E-04	1.6E-03	2.3E-03	1.3E-03	1.7E-03	2.1E-03
Traffic (Factor 8)	EC	μg/m^3	6.3E-01	2.7E-01	5.2E-01	7.1E-01	2.8E-01	5.2E-01	7.6E-01	4.9E-01	6.3E-01	7.7E-01
